# Summertime variability of the western North Pacific subtropical high and its synoptic influences on the East Asian weather

**DOI:** 10.1038/s41598-019-44414-w

**Published:** 2019-05-27

**Authors:** Woosuk Choi, Kwang-Yul Kim

**Affiliations:** 1 Seoul Institute of Technology, Seoul, 03909 Korea; 2 School of Earth and Environmental Sciences, Seoul National University, Seoul, 08826 Korea

**Keywords:** Atmospheric dynamics, Environmental impact, Climate-change impacts

## Abstract

Variation of the western North Pacific subtropical high (WNPSH) is an important meteorological factor for determining summertime rainfall and temperature over East Asia. Here, three major modes of summertime WNPSH variability are identified and corresponding environmental changes are investigated using cyclostationary empirical orthogonal function analysis. The leading mode exhibits a clear reinforcement of WNPSH associated with global warming. The second and third modes are characterized by intra-seasonal variation of the WNPSH intensity related to sea surface temperature variability in the central and eastern equatorial Pacific. Although WNPSH variability is regarded as a local manifestation, it reflects much wider changes in the entire North Pacific. The three modes exert seasonally and geographically distinct impacts on the East Asian weather by setting anomalous atmospheric circulation and altering the direction of moisture and heat transport. As such, the leading WNPSH modes are an important indicator of summertime weathers in countries neighboring the western North Pacific. This study also shows that extreme weather events are likely to increase as global warming intensifies.

## Introduction

Among the global-scale circulations, the Hadley circulation over the Pacific is the most dominant meridional air movement caused by strong upward motion with low-level convergence in the tropics due to tropical heat surplus^1,2^. The sinking branch of the Hadley circulation makes large and semi-permanent anticyclones over the mid-latitude Pacific^3–5^. Variation of this subtropical high is caused by changes in both tropical and mid-latitude environments, including tropical convective activity^6,7^, meridional circulations^1,8,9^, zonally asymmetric diabatic heating^3,10^, and synoptic wave activity in the extratropical region^11,12^. Generally, this anticyclone system intensifies in boreal summer, and it expands westward to the western North Pacific^13,14^. According to the extent to which the western North Pacific subtropical high (WNPSH) expands toward the East China Sea, significant difference is observed in monsoon circulation, typhoon tracks, and moisture transport over East Asia, shaping distinctive summer weathers in the coastal regions^3,15–17^.

Due to its importance in East Asian weathers, several studies have attempted to investigate WNPSH variability. Some studies used a critical value such as 5870 m geopotential height contour on the 500-hPa pressure surface as the location of the WNPSH boundary^13,16,18^. However, investigation of long-term WNPSH variation on the basis of this empirically determined criterion is difficult, because the thickness of atmospheric column keeps on increasing due to continued tropospheric warming. A robust rising trend of geopotential height makes it ambiguous to examine seasonal and inter-annual WNPSH variability based on the location of the 5870 m contour alone. As a simple alternative, zonal mean of geopotential height at a corresponding pressure level is extracted from the raw data, called eddy geopotential height^19–24^. This method can partially make up for the increasing trend of the atmospheric column thickness, but it would not accurately delineate WNPSH variability^25^. Not only is global warming signal itself an important climate factor for WNPSH variability, but also global warming effect may not necessarily be zonally uniform. Thus, dubiety is inexorable in conclusions based on a crude exclusion of global warming effect.

The objective of this study is to define major WNPSH variability without ambiguity and understand its physical linkage to East Asian summertime weathers in the context of large-scale climate dynamics. Previous studies have investigated how slowly-varying oceanic variability such as El Niño–southern oscillation or Pacific decadal oscillation affects East Asian weathers^14,26–30^. In the present study, however, WNPSH variability over the subtropical region is a focus of investigation so that East Asian weathers are understood directly in terms of WNPSH-induced atmospheric circulation changes rather than an indirect implication of ocean-atmosphere coupling processes. Distinct sources of WNPSH variability are separated and physically consistent atmospheric and oceanic patterns associated with them are identified via cyclostationary empirical orthogonal function (CSEOF) analysis^31,32^. By using this technique, key physical relationships between Pacific climate variability and WNPSH variation can be clearly identified. We, therefore, expect that this study would enable us to better understand WNPSH variability on various time scales and contribute to more accurate seasonal predictions of weather extreme events such as heavy rainfalls and heat waves during summer over East Asia.

## Results

### Variability of the WNPSH

**Figure 1:**
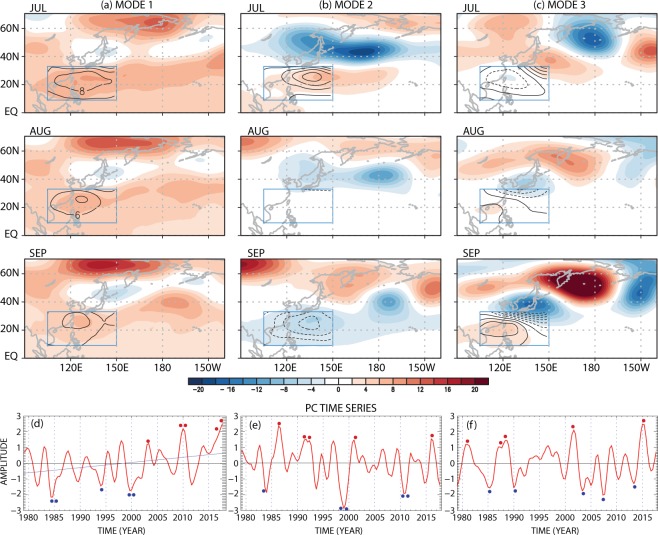
(**a**–**c**) Loading vectors of the leading three CSEOF (cyclostationary empirical orthogonal function) modes of 500-hPa geopotential height (m; black contour in the boxed target domain of 105°–150°E and 9°–32°N) and regressed patterns of monthly 500-hPa geopotential height (m; shades) over our analysis domain (90°–220.5°E, 0–70.5°N). (**d**–**f**) The corresponding PC (principal component) time series of the leading three CSEOF modes. Blue line in (**d**) represents a linear fit to the PC time series of the 1st mode. Red and blue dots indicate respectively the top and the bottom five years in the PC time series for composite analysis. This figure was created by using the Grid Analysis and Display System (GrADS) version 2.1 available at http://cola.gmu.edu/grads.

Three major modes of WNPSH variability during July through September are identified from 500-hPa geopotential height via CSEOF analysis (Fig. 1); the seasonal cycle is removed prior to CSEOF analysis. They explain 39%, 20%, and 11% of the total variability, respectively. We selected a small target area in the subtropics to isolate WNPSH variability (boxed region of 105°–150°E and 9°–32°N in Fig. 1). Atmospheric variability in mid-latitudes is generally far greater than that in the subtropics, since the former is influenced strongly and frequently by synoptic wave activities^33–35^. Therefore, WNPSH variability is analyzed by excluding the mid-latitude region.

The spatial patterns of the loading vector and corresponding time series showing an increasing trend indicate that the first mode is associated with widespread warming (Fig. 1a,d). Among many climatic influences, thickening of the atmospheric column due to natural and anthropogenic warming is identified as the most dominant mode of WNPSH variability. The regressed patterns of geopotential height over a wider analysis domain (90°–220.5°E, 0–70.5°N) also show clear positive values over much of the North Pacific (see the method section). Considering the trend in the PC time series (Fig. 1d), the atmospheric column has thickened by more than 15 m in the target area due to man-made warming during the record period (39 years). This implies that WNPSH is now stronger and is shifted more toward the Asian continent than in the past due to tropospheric warming (see the 5870 m contour in Fig. S1a,b). The interannual variability in the corresponding PC time series look similar to that of global sea surface temperature anomalies^36^, suggesting that the two variables have a common source of variability.

The second mode depicts intraseasonal variability of WNPSH with positive values in July but negative values in September (boxed region in Fig. 1b). This temporal contrast implies a westward extension and an eastward retreat of WNPSH on an intraseasonal time scale (see Fig. S1c). Based on the regressed patterns, this mode seems associated with a meridional shift of large-scale circulation during summer (Fig. 1b). As a result, circulation patterns change dramatically between the subtropical and high-latitude regions. The third mode also shows intraseasonal variation of mid-tropospheric geopotential height in the target domain (Fig. 1c). Given the regressed geopotential height patterns, this mode appears to be associated with tropical convection and resulting Rossby wave propagation toward North America^37–39^. In September, the anticyclonic circulation in the subtropics and the cyclonic circulation in the mid-latitude region are strengthened over the western Pacific^40^. There is no long-term trend in the second and the third CSEOF modes (Fig. 1e,f). By adding the loading vectors to the monthly climatology, substantial differences in the location of the WNPSH boundary can be appreciated among the three modes (see Fig. S1). Such differences will be shown to strongly modulate East Asian weathers.

### Composite analysis to WNPSH modes

Due to a significant difference in thermal inertia, notable pressure contrast develops between the continent and the ocean, which in turn drives strong atmospheric transport along the continental boundary^41–43^. According to the location of WNPSH with respect to the East Asian continental boundary, direction of moisture and heat transport may significantly vary. In this respect, location of the WNPSH boundary is a crucial factor for modulating summertime weathers in the East Asian countries. Among various weather phenomena, summertime heavy rainfalls and heat waves are most critical to human lives. In order to examine how each WNPSH mode affects summertime weathers, composite analysis is carried out by selecting the highest and lowest five years from the PC time series in Fig. 1 (see Table S1 for the selected years). Difference between these two groups represents the meteorological impacts of each mode to summer weathers in East Asia.

**Figure 2:**
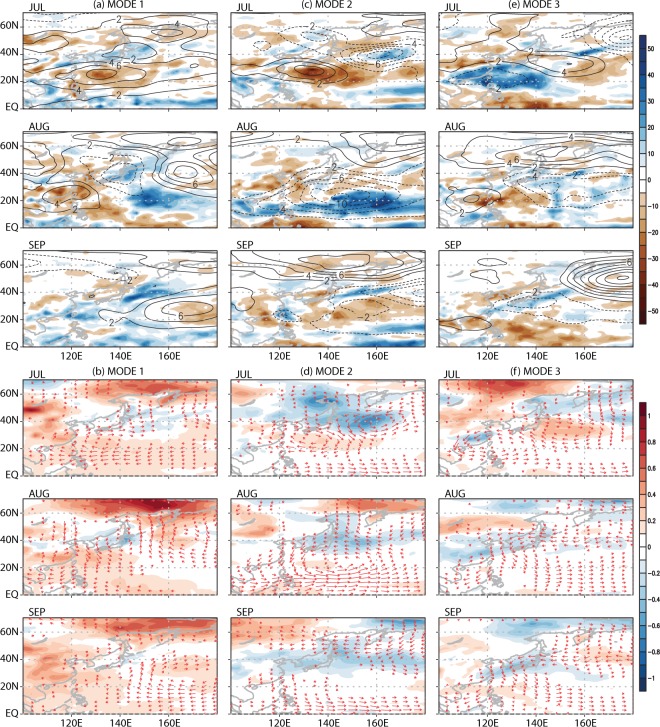
Composite differences between five positive and negative years of (upper panel) precipitation (shade; mm) and 850-hPa geopotential height (contour; m), and (lower panel) surface temperatures (shade; °C) and 850-hPa wind (arrow) for the three WNPSH CSEOF modes. This figure was created by using the Grid Analysis and Display System (GrADS) version 2.1 available at http://cola.gmu.edu/grads.

Figure 2 shows the composite patterns based on the difference between the two groups mentioned above. To facilitate the interpretations, composite patterns of 850-hPa geopotential height are overlaid on the precipitation patterns, whereas composite patterns of 850-hPa horizontal wind are overlaid on top of the surface temperature patterns. Overall, amount of precipitation is reduced at the center of high-pressure anomalies while increased precipitation is observed at the center of low-pressure anomalies and along the perimeter of the high-pressure center in years of the positive phase (Fig. 2a,c,e). Precipitation changes are prevalent over the ocean and the subtropical coastal regions. In general, surface (2 m) warming (cooling) is seen in association with high (low) pressure anomalies (Fig. 2b,d,f). Temperature changes tend to be larger over the continent than over the ocean, and largest warming is observed typically in high-latitude areas. It is remarkable that WNPSH variations in a small area result in fairly broad and distinct impact all over the western North Pacific.

### Weather responses to the three WNPSH modes

**Figure 3:**
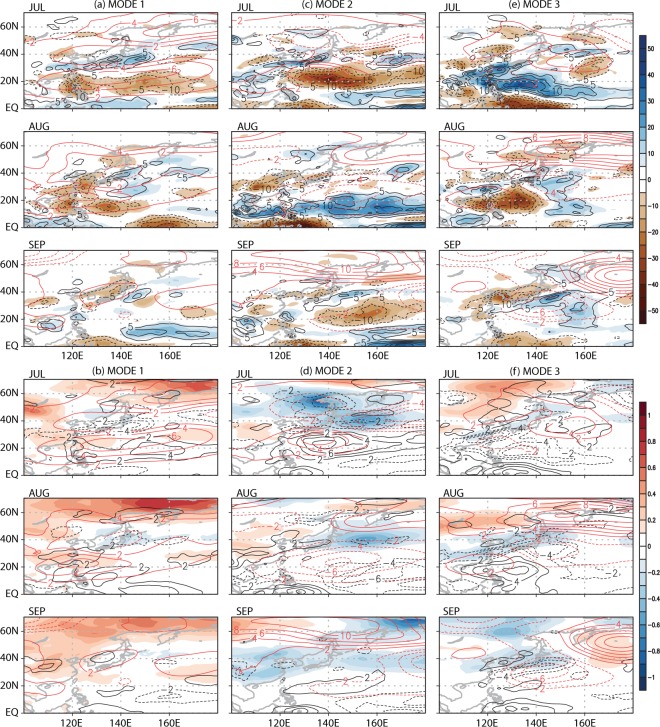
The monthly-averaged regressed patterns of daily predictor variables for the three WNPSH modes: (upper panel) precipitation (shade; mm), 1000–500 hPa vertical moisture convection (contour; g kg^−1^), and 850-hPa geopotential height (red contour; m), and (lower panel) surface (2 m) air temperature (shade; °C), net radiation at the surface (contour; W m^−2^) and 850-hPa geopotential height (red contour; m). This figure was created by using the Grid Analysis and Display System (GrADS) version 2.1 available at http://cola.gmu.edu/grads.

The composite analysis above determines the meteorological difference between the years of high and of low PC amplitudes from the raw data. In this section, physical mechanisms responsible for these weather changes will be examined by using regression analysis in CSEOF space (see the methods section). Daily datasets, unless otherwise stated, are used in order to address both the mean and variance of physical changes in each month. Figure 3 shows the regressed patterns of precipitation and temperature for the leading WNPSH modes. Physically consistent variations of 850-hPa geopotential height, monthly total vertical moisture convection averaged over 1000–500 hPa, and net radiation at the surface are also depicted in order to understand the mechanisms of how the WNPSH modes affect summertime weathers (see also other variables in Figs S2 and S3). Overall, the regressed patterns of precipitation and surface air temperature in Fig. 3 are similar to the composite patterns in Fig. 2. The three WNPSH modes show unique characteristics in modulating tropospheric circulation, resulting in seasonally distinctive impacts on East Asian weathers.

Precipitation associated with the first WNPSH mode exhibits distinct behavior for each month (Fig. 3a). In July, precipitation decreases in the subtropical region including the Philippines, and increases zonally from East China to Japan. This pattern seems consistent with the intensification of WNPSH and the extension of its boundary toward the Asian continent. As the WNPSH boundary approaches closer to the continent during August, precipitation decreases significantly over the coastal areas. In September, the WNPSH boundary penetrates the continent and negative precipitation anomaly almost disappears near Taiwan but remains over Korea and Japan. These anomalous precipitation patterns are well explained in terms of the change in vertical moisture convection (Fig. 3a), which is closely related to the location of the WNPSH boundary (Figs 1a and S1b). Near the center of an anticyclonic circulation, sinking motion prevails, whereas just outside an anticyclonic circulation, there is a general rising motion (see Fig. S2a). As the anticyclone approaches the continent, therefore, moisture convection weakens significantly leading to decreased precipitation in the coastal regions of the Asian continent. In September, 5870 m geopotential height contour penetrates deep into the continent (Fig. S1b), resulting in westward transport of tropical moisture. This implies that moisture transport from the tropical Pacific to East Asia weakens significantly.

The first WNPSH mode represents consistent warming over China and the high-latitude region during the whole summer (Fig. 3b). Major surface warming is observed over much of the East Asian continental region but not over the tropical ocean. It seems that decreased total cloud cover and the resulting increase in net radiation at surface are the primary factors explaining the surface warming (Figs 3b and S3a). Total cloud cover and net surface radiation, in turn, are closely related to the 850-hPa geopotential height change (Fig. 3b). Over the center of an anticyclonic circulation, cloudiness decreases and net radiation increases. Situation reverses for a cyclonic circulation. Significant warming is observed over the high-latitude region, which is consistent with the well-known characteristic of global warming^44^. In general, temperature response is more obvious over the continent due to the lower heat capacity whereas precipitation changes are more notable over the ocean.

For the second mode, precipitation changes match well with the WNPSH variations and are largely explainable by vertical moisture convection (Fig. 3c). In July, reduced precipitation due to a stronger WNPSH is observed in the Taiwan and the Philippine Seas (Fig. S1c). As WNPSH shifts slightly northward from its normal position in August, increased precipitation is seen in the tropical region of the Pacific. No significant precipitation change is seen in the coastal regions of East Asia in September, when WNPSH moves away from the continent. In addition, surface temperature exhibits warming in the China mainland during July to August but cooling in September (Fig. 3d). These differences are explained in terms of net radiation change and heat transport associated with the location of the WNPSH boundary (i.e., expansion in July and retreat in September). Throughout the summer, consistent and robust cooling is observed over Japan and the North Pacific due to the persistent cloudy conditions and net radiation deficit associated with the low-pressure anomalies (Fig. 3d).

The third mode shows a notable increase of precipitation in the coastal region of East Asia in July, when WNPSH is weaker and further away from the continent (Figs 1c and S1d). As WNPSH shifts southward from its normal position in August, significant reduction in precipitation is observed in the subtropics (Fig. 3e). In September, decreased rainfall is seen over the eastern China, Korea, and Japan as WNPSH penetrates deep into the continent and, as a result, moisture transport from the subtropics to mid-latitudes weakens significantly. The spatial pattern of precipitation is again closely linked with the location and intensity of WNPSH (i.e., retreat in July and expansion in September; see Fig. S1d) and is explained reasonably in terms of vertical moisture convection. Surface cooling is found over East China, Korea, and Japan during the whole summer (Fig. 3f). This cooling is mainly due to the reduced net radiation at surface and partly to cold advection from the north (Fig. S3c). Notable net radiation change in the tropical ocean, however, is not readily reflected in temperature due to the large ocean heat capacity.

Regression analysis in CSEOF space explains well the physical relationship between the East Asian summer weathers and the WNPSH variability. As can be seen in Fig. 3 (and also Figs S2 and S3), changes in precipitation and temperature for each WNPSH mode are reasonably explained in terms of changes in circulation, vertical motion, moisture convection, net surface radiation, cloudiness among others. In addition, variance of daily precipitation and daily surface temperature changes associated with the three WNPSH modes can be assessed quantitatively (Fig. S4). If the daily variance is considered together with the monthly mean, each of the leading WNPSH modes may result in significant variations of rainfall and temperature; up to ~70–80 mm (~30%) of rainfall variation and temperature fluctuations of up to ~1.5–2 °C based on the 2-sigma (standard deviation) values. This suggests that the leading WNPSH modes can cause extreme weather conditions during summer.

## Discussion

**Figure 4:**
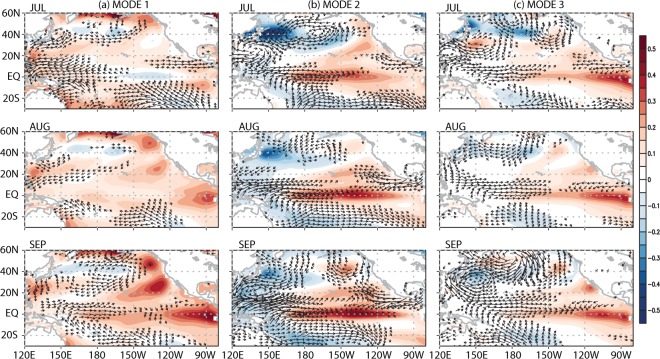
The regressed pattern of monthly sea surface temperature (shades; °C) and monthly horizontal wind at the surface (arrow; m s^−1^) for the three WNPSH modes during July–September. This figure was created by using the Grid Analysis and Display System (GrADS) version 2.1 available at http://cola.gmu.edu/grads.

Variation of WNPSH is an important modulator of East Asian weathers on daily and intra-seasonal time scales via a direct control of the atmospheric circulation in the North Pacific and East Asia. The distinct sources and physical mechanisms of the three leading modes of WNPSH variability are investigated in the present study. The three leading modes all seem physically related with large-scale sea surface temperature (SST) variability in the Pacific (Fig. 4). It seems that the tropical western Pacific responds rather quickly to SST change in the equatorial eastern Pacific through the oceanic and atmospheric dynamics^23,45–47^. Specifically, the regressed patterns of SST suggest that the second and the third WNPSH modes are linked with the central Pacific (CP) and the eastern Pacific (EP) El Niño events, respectively. It is of interest to note that the intraseasonal WNPSH variability as represented by these modes is intimately connected with the seasonally persistent SST variability in the equatorial Pacific. Despite the seemingly similar forcing (i.e., SST) patterns in summer, seasonal evolution of WNPSH varies significantly in terms of its intensity and location from one mode to another. In future study, it would be important to delineate physical linkage between WNPSH variability and SST variability such as El Niño-southern oscillation and Pacific decadal oscillation.

This study shows that the effect of global warming and El Niño, although it is prominent in winter, is not limited to winter. Global warming and El Niño appear to affect the location and intensity of WNPSH significantly in summer by altering the distribution of energy and subsequent circulation in the tropics. The summertime precipitation and surface temperature in East Asia, in turn, are affected considerably by the variation of WNPSH. It is remarkable that the variation of the semi-permanent subtropical Pacific anticyclone in a small area of the western Pacific (boxed domain in Fig. 1) exerts significant impacts on summertime weathers not only along the coast of the Asian continent but also over a much wider Pacific region from the tropics to high-latitudes. This indicates that monitoring of WNPSH is an important issue for forecasting weathers in the countries around the western North Pacific.

The warming mode, which was often excluded in other studies, turns out to exert strongest influence on the intensity and location of WNPSH. The continental areas tend to become warmer in summer (JAS) and the coastal regions experience drier condition in August in East Asia. The present study strongly suggests a possibility that inclement weathers will become more frequent in the East Asian countries unless global warming is restrained by policy efforts.

## Methods

### Reanalysis datasets

We used 39-year (1979–2017) European Center for Medium-Range Weather Forecast ERA-interim reanalysis datasets at a horizontal resolution of 1.5° × 1.5°^48^. Target variable is chosen to be the monthly 500-hPa geopotential height to diagnose the multi-time scale variability of the WNPSH. The daily mean oceanic and atmospheric variables such as SST, 850-hPa geopotential height, surface (2 m) temperature, 3-dimensional wind, net radiation at the surface, precipitation, specific humidity, total cloud cover, total column water, and relative humidity were used to investigate the synoptic influences of WNPSH to East Asian weather.

### Cyclostationary Empirical Orthogonal Function analysis

The CSEOF analysis technique is used to extract major modes of the summertime WNPSH variability^31,32^. In this analysis, meteorological datasets which have a dimension of both space and time, *T*(*r*, *t*), can be written as

1 $$\begin{array}{cc}T(r,t)=\sum _{n}\,L{V}_{n}(r,t)P{C}_{n}(t), & r\in D,\,t\in T\end{array}.$$

where *n* is the CSEOF mode number, *LV*_*n*_(*r*, *t*) is called the *n*th CSEOF loading vector, and *PC*_*n*_(*t*) is corresponding PC time series, *r*, and *t* stand for space and time, and *D* and *T* denote the spatial domain and the record period for CSEOF analysis, respectively. A major difference from the traditional EOF analysis is that CSEOF analysis captures temporally varying spatial patterns. CSEOF loading vectors are temporally evolving but repeating spatial patterns, i.e.,

2 $$L{V}_{n}(r,t)=L{V}_{n}(r,t+d),$$

where *d* is called the nested period. In the present study, nested period is set to three months because we focus on the boreal summer (i.e., JAS); we chose JAS since 500-hPa geopotential height over the target (WNPSH) area are maximized in these three months. CSEOF loading vectors are mutually orthogonal in space and time, and PC time series are mutually uncorrelated.

To understand the environmental responses to the variation of 500-hPa geopotential height, regression analysis is conducted in CSEOF space^49^. The evolutions of atmospheric and oceanic variables including SST, precipitation, 2 m temperature, and all other analysis variables are decomposed to be physically consistent with those of 500-hPa geopotential height (target variable in this study) via regression analysis in CSEOF space. The key idea of regression in CSEOF analysis is that all the variables are decomposed to have the identical PC time series of the target variable. To accomplish this goal, PC time series of the predictor variable, *PCP*_*m*_(*t*), is regressed onto the target PC time series, *PC*_*n*_(*t*), as in (3):

3 $$P{C}_{n}(t)=\sum _{m=1}^{M}\,{a}_{m}^{(n)}PC{P}_{m}(t)+{\varepsilon }^{(n)}(t),\,n=1,2,\cdots ,$$

where *M*(=20), ${a}_{m}^{(n)}$, and *ε*^(*n*)^ denote the number of predictor PC time series used for regression, regression coefficients for mode *m*, and regression error time series, respectively. If the temporal resolutions of the two sets of PC time series are not the same, one set of time series (typically time series with a higher temporal resolution) should be interpolated to match the temporal resolution of the other variable in (3). Then, regressed CSEOF loading vectors for the predictor variable, *LVPR*_*n*_(*r*, *t*), are obtained as in (4):

4 $$LVP{R}_{n}(r,t)=\sum _{m=1}^{M}\,{a}_{m}^{(n)}LV{P}_{m}(r,t),\,r\in R,\,t\in T,$$

where *LVP*_*m*_(*r*, *t*) are the CSEOF loading vectors derived from the predictor variable. Note that the regressed patterns in (4) are defined over the spatial domain *R*, which may not necessarily be the same as the target domain *D* in (1). Further, the temporal resolutions of the target and predictor variables differ in this study. For example, the patterns in Fig. 3 are the monthly averages of daily regressed loading vectors over a domain much wider than the target area. Entire variables used in this study, then, can be written as

5 $$Data\,(r,t)=\sum _{n}\,\{{T}_{n}(r,t),\,{R}_{n}(r,t),\,{U}_{n}(r,t),\,{V}_{n}(r,t),\,\ldots \}P{C}_{n}(t),$$

where $\{{R}_{n}(r,t),\,{U}_{n}(r,t),\,{V}_{n}(r,t),\cdots \}$ are referred to as the regressed CSEOF loading vectors of predictor variables which are regarded to be physically consistent with the *n*th mode of the target variable, *T*_*n*_(*r*, *t*).

## Supplementary information

Supplementary information

## Data Availability

The data that support the findings of this study are available from the corresponding author (KYK) upon reasonable request.
