# Supplementary material for: Summertime variability of the western North Pacific subtropical high and its synoptic influences on the East Asian weather
Source: Sci Rep. 2019 May 27;9:7865. doi: 10.1038/s41598-019-44414-w (PMC6536516; doi:10.1038/s41598-019-44414-w)
Supplement: Supplementary file 1 — Supplementary information [file 41598_2019_44414_MOESM1_ESM.pdf]

Summertime variability of the western North Pacific subtropical high and its synoptic influences on the East Asian weather

Woosuk Choi

Seoul Institute of Technology, Seoul, Korea

Kwang-Yul Kim*

School of Earth and Environmental Sciences, Seoul National University, Seoul, Korea

Supplementary information

Table S1. The highest and lowest five years of PC time series for each mode.

Mode	Highest five years	Lowest five years
Mode 1	2003, 2009, 2010, 2016, 2017	1984, 1985, 1994, 1999, 2000
Mode 2	1986, 1991, 1992, 2001, 2016	1983, 1998, 1999, 2010, 2011
Mode 3	1981, 1987, 1988, 2001, 2015	1985, 1990, 2003, 2007, 2013

This table shows the years of the highest five and the lowest five peaks in the PC time series of the first three CSEOF modes in Figures 1d-e in the text. Composite analysis in Figure 2 in the main text is conducted by calculating averages for the selected five years.

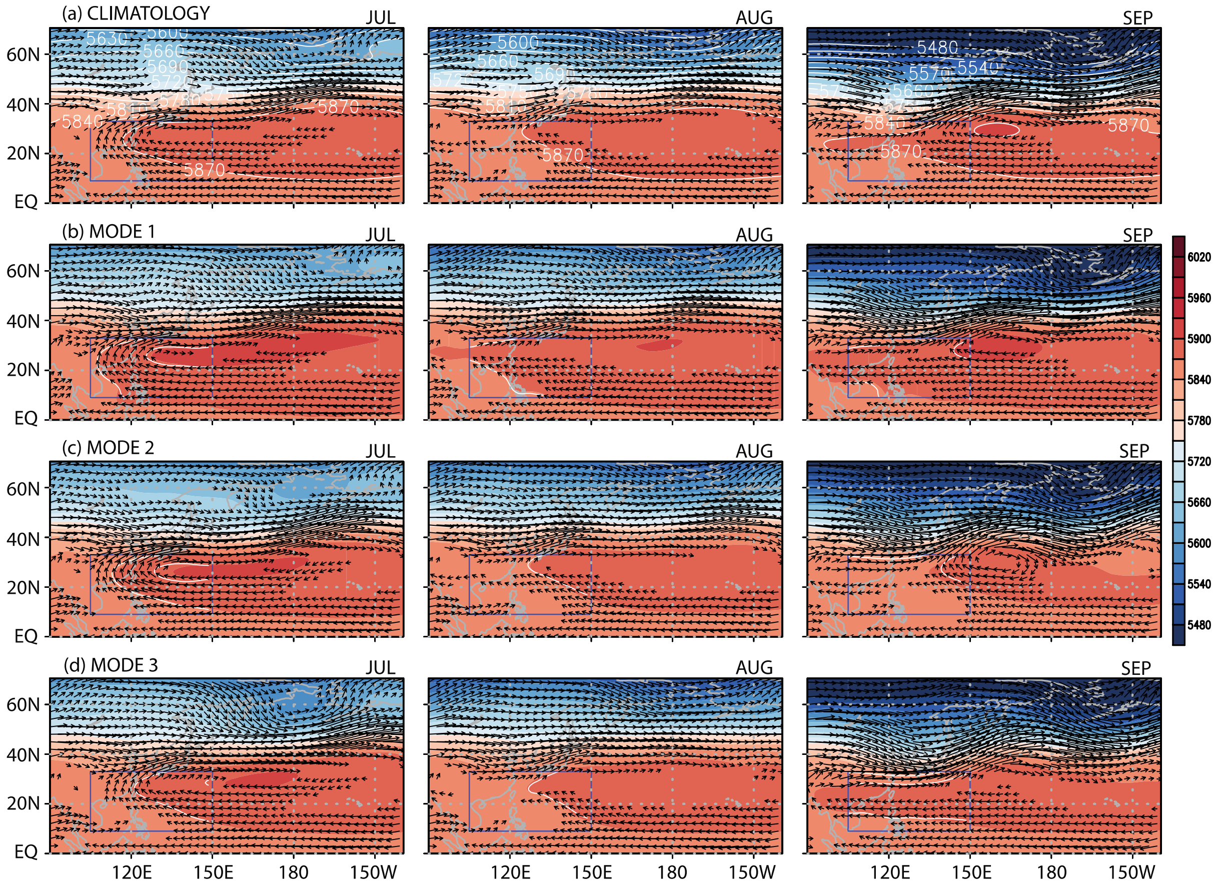

Figure S1. (a) Monthly climatology and (b–d) two times the regressed loading vectors of monthly 500-hPa geopotential height and horizontal wind during July–September superposed on the climatology. The white line in the boxed domain of 105°–150°E and 9°–32°N represents two times the WNPSH loading vectors added to the monthly climatology, showing the approximate position of the WNPSH boundary on the 500-hPa pressure surface. The factor of two is used to show the maximum displacement according to the PC time series in Figure 1. This figure was created by using the Grid Analysis and Display System (GrADS) version 2.1 available at http://cola.gmu.edu/grads.

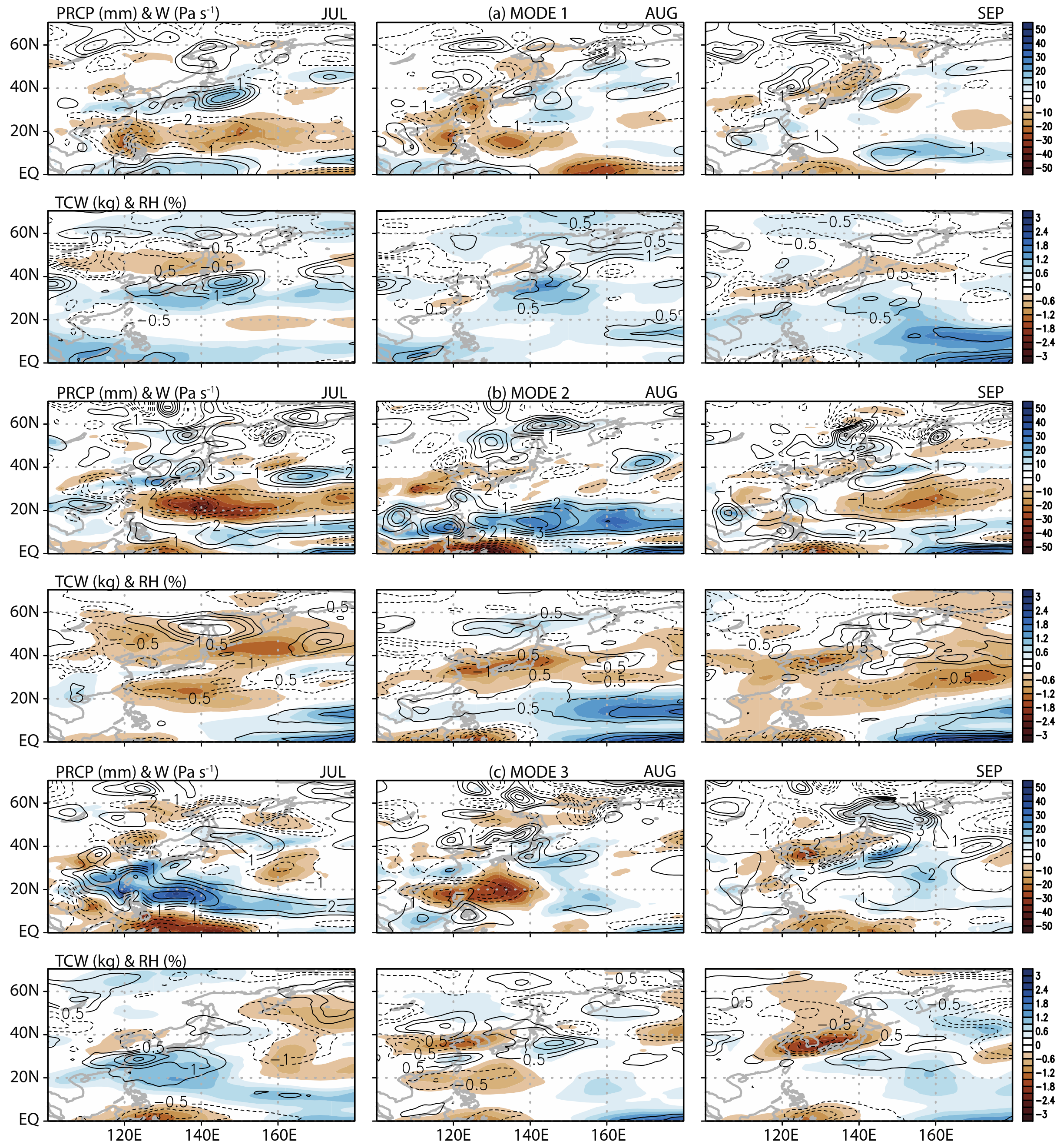

Figure S2. The monthly averaged regressed patterns of daily predictor variables for the three WNPSH modes: precipitation (shade; mm), vertical velocity (contour; –Pa s^{-1}), total column water (shade; kg), and relative humidity (contour; %). This figure was created by using the Grid Analysis and Display System (GrADS) version 2.1 available at http://cola.gmu.edu/grads.

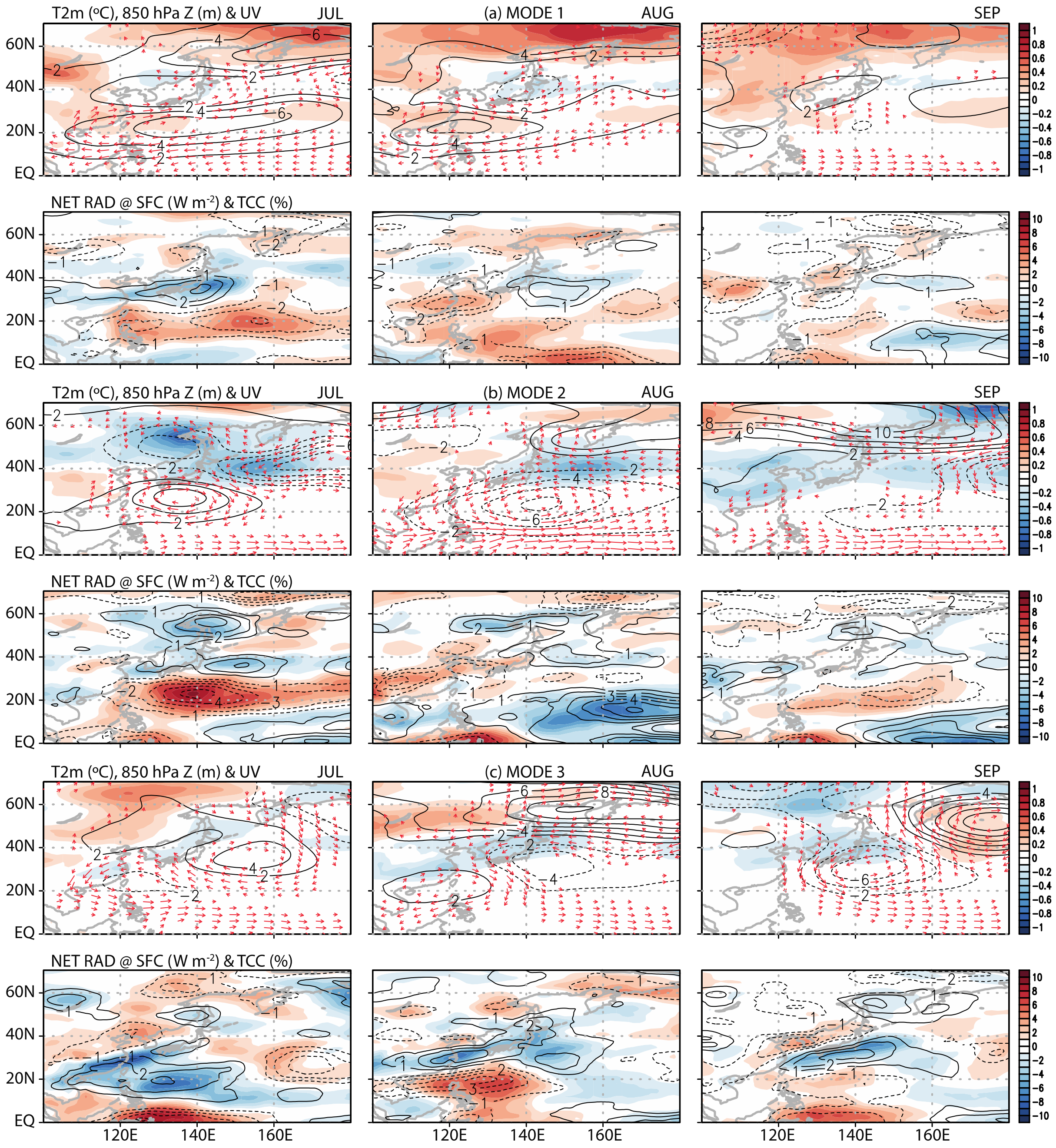

Figure S3. The monthly-averaged regressed patterns of daily predictor variables for the three WNPSH modes: surface (2 m) air temperature (shade; °C), 850-hPa geopotential height (contour; m), 850-hPa surface wind (vector), net radiation at surface (shade; W m^{-2}), and total cloud cover (contour; %). This figure was created by using the Grid Analysis and Display System (GrADS) version 2.1 available at http://cola.gmu.edu/grads.

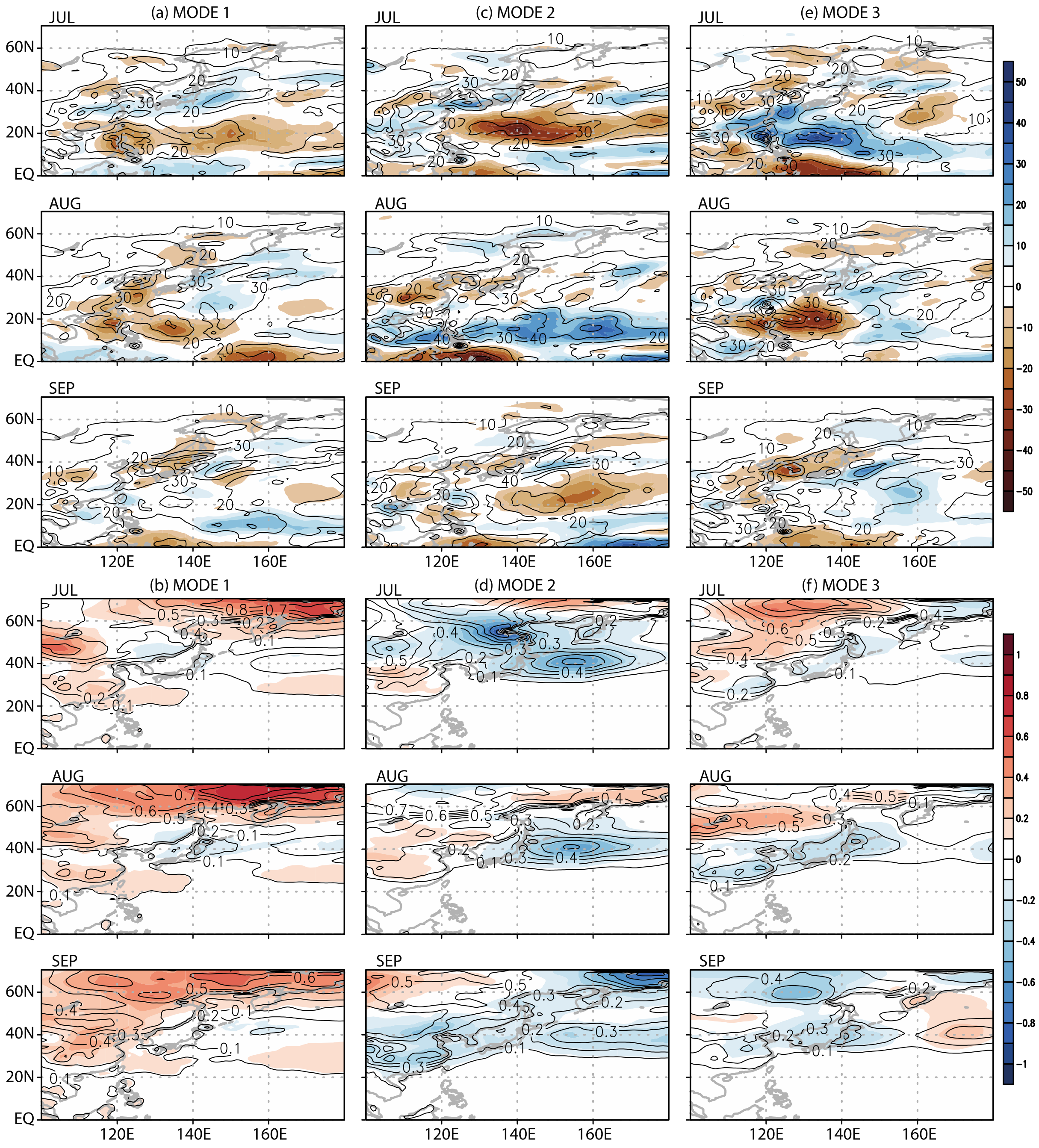

Figure S4. The monthly-averaged regressed patterns of (upper panel) daily total precipitation (shades; mm) and (lower panel) daily-mean anomalous surface (2 m) air temperature (shades; °C) for the three WNPSH modes. Standard deviations (contours) are derived from the daily regressed loading vectors for each month. This figure was created by using the Grid Analysis and Display System (GrADS) version 2.1 available at http://cola.gmu.edu/grads.
